# Increased antibiotic resistance of *Pseudomonas aeruginosa* isolates from chronic rhinosinusitis patients grown in anaerobic conditions

**DOI:** 10.1002/lio2.1244

**Published:** 2024-05-09

**Authors:** Jannatul Ferdoush Tuli, Mahnaz Ramezanpour, Clare Cooksley, George Spyro Bouras, Kazuhiro Ogi, Sholeh Feizi, Roshan Nepal, Alkis James Psaltis, Peter‐John Wormald, Sarah Vreugde

**Affiliations:** ^1^ Department of Surgery–Otolaryngology, Head and Neck Surgery University of Adelaide Adelaide South Australia Australia; ^2^ Basil Hetzel Institute for Translational Health Research, Central Adelaide Local Health Network, The Queen Elizabeth Hospital Woodville South South Australia Australia; ^3^ Division of Otorhinolaryngology Head and Neck Surgery, Department of Sensory and Locomotor Medicine, Faculty of Medical Sciences University of Fukui Fukui Japan; ^4^ CSIRO Marine and Atmospheric Research The Commonwealth Scientific and Industrial Research Organisation (CSORO) Hobart Tasmania Australia

**Keywords:** anaerobic conditions, antibiotic, chronic rhinosinusitis, pseudomonas aeruginosa, resistance

## Abstract

**Introduction:**

In chronic rhinosinusitis (CRS), the congestion and blockage of the nose can cause anaerobic conditions within the sinus cavities which may promote the expression of virulence and antibiotic resistance genes in invading pathogens. *Pseudomonas aeruginosa* is a facultative anaerobic bacteria and causes severe recalcitrant CRS. In this study, we aimed to evaluate the antimicrobial resistance of *P. aeruginosa* isolates of CRS patients in planktonic and biofilm form grown in aerobic and anaerobic conditions.

**Methods:**

*P. aeruginosa* clinical isolates of CRS patients (*n* = 25) were grown in planktonic and biofilm form in aerobic and anaerobic conditions. Minimum inhibitory concentrations (MIC) of planktonic forms and minimum biofilm eradication concentrations (MBEC) were determined. Additionally, metabolic activity by fluorescein diacetate assay, biofilm biomass by crystal violet assay and eDNA concentration were assessed in both conditions.

**Results:**

*P. aeruginosa* planktonic cells grown in anaerobic condition exhibited increased gentamicin resistance (*p* < .01), whereas *P. aeruginosa* biofilms grown in anaerobic condition displayed significantly increased MBEC values for gentamicin (*p* < .0001) and levofloxacin (*p* < .001). The metabolic activity of anaerobic biofilms was significantly higher compared with aerobic biofilms (*p* < .0001). However, the biofilm biomass of isolates grown in aerobic conditions was higher than anaerobic conditions (*p* < .5).

**Conclusion:**

*P. aeruginosa* isolates from CRS patients grown in anaerobic conditions showed significantly increased resistance to antibiotics with an increased metabolic activity but decreased biofilm biomass.

**Level of Evidence:**

NA.

## Graphical abstract

*P. aeruginosa* isolates from CRS patients grown in anaerobic conditions showed significantly increased tolerance to antibiotics with an increased metabolic activity but decreased biofilm biomass.

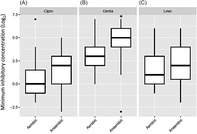

## 1 INTRODUCTION

In chronic rhinosinusitis (CRS), inflammation of the paranasal sinus mucosa can create congestion and blockage of the sinus ostia, leading to the development of hypoxic conditions within the sinus cavities. Studies have indeed demonstrated hypoxia in the sinuses by measuring the oxygen tension with a digital monitoring system in CRS patients.^1^ *Pseudomonas aeruginosa* is the second most common bacterium isolated in CRS^2^ and its presence is associated with severe recalcitrant CRS.^3^ *P. aeruginosa* is a facultative anaerobe that can proliferate in the presence of oxygen and also in the absence of oxygen with nitrate (NO_3−_) or nitrite (NO_2−_) as a terminal electron acceptor.^4^ Along with the free‐form planktonic lifestyle of *P. aeruginosa*, the bacteria can form dense communities encased in an exopolysaccharide matrix known as biofilm.^4^, ^5^, ^6^, ^7^, ^8^ Biofilm‐producing bacteria are responsible for recurrent nosocomial infections and chronic infectious diseases, resulting in prolonged hospital stays and increased morbidity and mortality.^9^ They exhibit greater antibiotic tolerance compared to planktonic bacteria.^9^ Several factors contribute to the increased resistance of biofilms to antibiotics including slow growth and metabolism, limited oxygen penetration and reduced susceptibility of dormant bacteria deep within biofilms.^5^ Biofilms also form a penetration barrier based on binding of positively charged aminoglycosides to the negatively charged alginate polymers, the presence of b‐lactamase from the bacteria, which cleaves and traps b‐lactam‐antibiotics and overexpression of efflux pumps.^6^, ^7^, ^8^ Therefore, biofilm elimination is crucial for the eradication of chronic infections in diseases like CRS.

CRS is ubiquitous in patients with cystic fibrosis (CF), accounting for significant morbidity and contributing to CF lung disease.^10^ On CT scan or clinical assessment, over half of adult CF patients have sinusitis recurrence and almost 100% have sinus disease.^11^ As a result of excessive mucus in the lungs and sinuses coupled with a reduced mucociliary function, CF patients are more vulnerable to chronic relapses of infection and inflammation. In early disease, both the sinuses and lungs can be infected with bacteria such as *Staphylococcus aureus*, *Haemophilus influenzae* and *Streptococcus pneumoniae*, whereas *Pseudomonas aeruginosa* is a major pathogen in later stage disease. High mortality rates are observed in CF patients with *P. aeruginosa* airway infections.^8^ Studies have shown the ability of *P. aeruginosa* to grow and establish drug resistant biofilms in the lungs of CF patients by the hypersecretion of a viscous mucus layer in the CF airway, which provides a low oxygen environment, and the presence of extracellular DNA (eDNA).^8^, ^12^

Apart from two older studies on the susceptibility of *P. aeruginosa*'s under aerobic and anaerobic conditions,^13^, ^14^ there is a notable lack of literature in this area. Here, we examined the effects of anaerobic growth on the susceptibility to antibiotics of *P.aeruginosa* clinical isolates from CF and non‐CF CRS patients from different geographic locations. We also investigated the effect of anaerobic conditions on the metabolic activity, biofilm biomass and biofilm eDNA concentrations.

## 2 METHODS AND MATERIALS

### 2.1 Clinical isolates and Bacterial growth

The study was performed with the recommendation of Queen Elizabeth Hospital of The Central Adelaide Local Health Network Human Research Ethics Committee (CALHN HREC) (HREC/18/CALHN/69) with written informed consent obtained from all patients. All samples were coded and anonymized before use. In total, 25 *P. aeruginosa* clinical isolates 11 from Australia, 7 from The Department of Otorhinolaryngology, Academic Medical Centre (Amsterdam, Netherlands) and 7 from the Department of Otolaryngology‐Head and Neck Surgery, University of Alabama at Birmingham (Birmingham, AL, USA) were collected from the sinonasal cavities of CRS patients (with and without CF) using endoscopically‐guided sinus swabs at the time of endoscopic sinus surgery from the inferior turbinate surface. The diagnostic criteria for CRS patients was contented by the American Academy of Otolaryngology and Head and Neck Surgery and the European Position Statement (EPOS).^15^

Bacterial glycerol stocks were prepared in Tryptone Soya Broth (TSB) media (OXOID Ltd., Basingstoke, Hampshire, England, UK) and stored at −80°C. Overnight cultures were prepared before the assay by subculturing bacterial strains onto fresh Tryptone Soya Agar (TSA) plates and incubating at 37°C overnight. For aerobic conditions, standard TSA plates were used and for anaerobic growth, TSA plates were supplemented with 1.5% KNO_3_. In anaerobic conditions, plates were incubated in an anaerobic gas jar with AnaeroGen sachets (Oxoid Limited, Basingstoke, UK). The gas composition inside the anaerobic chamber was 6%–13% of carbon dioxide and 1% of oxygen. Dry anaerobic indicator strips (Oxoid Ltd., Basingstoke, UK) were used to confirm the generation of anaerobic atmosphere inside the jar.

### 2.2 Planktonic and biofilm growth in aerobic and anaerobic conditions

In planktonic conditions, growth curves were established for each strain to ascertain the time taken to stationary phase of growth under aerobic (TSB medium) and anaerobic (TSB medium +1.5% KNO3) conditions at 37°C with agitation. Biofilms were grown in 6‐well (eDNA quantification) or 96‐well (MBEC, Crystal violet, FDA assay) plates in aerobic and anaerobic conditions using an inoculum from an agar plate with the adjusted opacity of MacFarland 1.0 ± 0.1 as previously described.^10^, ^11^ Biofilm plates were incubated for 48 h on a rotating platform (3D Gyratory Mixer; Ratek Instruments, Boronia, Australia) at 70 rpm.

### 2.3 Antibacterial agents

Gentamicin, ciprofloxacin and levofloxacin were used in this study (all from Sigma‐Aldrich, North Ryde BC, NSW, Australia). Gentamicin and ciprofloxacin were prepared in 0.1 N hydrochloric acid (Sigma‐Aldrich) and levofloxacin in 30% acetic acid (Ajax Finechem, Taren Point, NSW, Australia) then diluted in water. Storage temperature for all antibiotic stocks was −20°C until further use. For minimum inhibitory concentration (MIC), all tested antibiotic concentrations were prepared in sterile microtiter plates containing TSB medium.

### 2.4 Determination of Minimum Inhibitory Concentration (MIC)

To determine the MIC, the micro dilution method was used according to Wiegand et al.^16^ Bacterial clinical isolates were grown overnight on nutrient agar plates and were suspended in 0.9% normal saline solution at 0.5 McFarland Unit (MFU). The bacterial suspension was diluted 1:100 in TSB medium then 50 μL was added into a 96‐well plate (Costar, Corning Incorporated, Corning, USA) containing 50 μL of serially diluted antibiotics (concentration range 0.125–128 μg/mL). The microtiter plate was incubated at 37°C for 18–24 h before visual inspection for growth. The optical density at 595 nm was also read using a Bio‐Rad plate reader (BIO‐RAD Laboratories, CA, USA). A positive growth control containing untreated bacterial suspension and a negative control containing TSB alone were included.

### 2.5 Determination of Minimum Biofilm Eradication Concentration (MBEC)

Susceptibility assays in microtiter plates were carried out as described.^17^ Bacterial clinical isolates were cultured on TSA plates, and isolated single colonies in 0.9% saline were adjusted to 1.0 ± 0.1 McFarland units (approximately 3 × 108 CFU/mL). Then 150 μL of 1:15 bacterial suspension was added to wells in a 96‐well plate and incubated for 48 hours at 37°C on a rotating platform (3D Gyratory Mixer, Ratek Instruments, Boronia, Australia) at 70 rpm. After washing once with 1×PBS, 180 μL of antibiotics (ciprofloxacin, levofloxacin and gentamicin) were added serially diluted at a concentration of 1–128 μg/mL for aerobic and 1–256 μg/mL for anaerobic conditions. Plates were incubated at 37°C for 24 h to determine the MBEC (minimum biofilm eradication concentration) of each antibiotic. After washing once with 200 μL PBS, crystal violet staining was carried out. All eradication experiments were performed as two technical and three biological replicates.

### 2.6 Metabolic activity assay

The fluorescein diacetate (FDA) (Sigma‐Aldrich) assay was chosen as it can be utilized under both aerobic and anaerobic conditions. FDA was dissolved in acetone to a concentration of 10 mg/mL as described.^18^ Biofilms were rinsed once with 100 μL of fresh MOPS (3‐(N‐Morpholino) propane sulfonic acid) followed by the addition of 100 μL FDA working solution (1:50 dilution of stock). Plates were incubated in the dark at 37°C for 4 h and fluorescence measured on a plate reader (FLUOstar Optima, BMG Labtech, Ortenberg, Germany) at excitation 495 nm and emission 518 nm.^18^ Average fluorescence signals were plotted as a function of the incubation time to determine the optimal incubation period for each isolate.

### 2.7 Crystal violet assay

The crystal violet assay was used to determine the biomass of bacterial biofilms. Single bacterial colonies were suspended in 0.9% saline and adjusted to 0.30 McFarland units. The bacterial suspension was then diluted 1:15 in TSB medium and 150 μL of the diluted bacterial suspension was added to 96‐well microtiter plates (Costar, Corning Incorporated, Corning, USA). Plates were incubated at 37°C for 24 h on a rotating platform (3D Gyratory Mixer, Ratek Instruments, and Boronia, Australia) at 70 rpm. For anaerobic conditions, plates were incubated inside an Anaerogen pouch with an Anaerogen compact sachet. After 48 h, biofilms were rinsed once with PBS and the cells were stained with Crystal Violet (0.1% in water). The plates were then rinsed with distilled H_2_O and air dried completely overnight. Crystal violet was solubilized by adding 30% acetic acid and incubating on a plate shaker for 1 hour. Absorbance was measured at 595 nm.

### 2.8 Extracellular DNA (eDNA) quantitation assay

eDNA quantification was carried out as described^19^ with some minor modifications in biofilm growth. In brief, biofilms were grown in 6‐well plates (Greiner Bio‐One, Kremsmünster, Austria) in aerobic and anaerobic conditions using an inoculum from an agar plate with the adjusted opacity of MacFarland 1.0 ± 0.1 then diluted in TSB media as 1:15. After 48 h growth, biofilms were washed once with PBS without disturbing the adherent film. 500 μL of rehydration solution (Promega, Madison, WI, USA) was added and the plates mixed by vortexing to disperse the biofilm. The suspension was transferred from the 6‐well plate to an Eppendorf tube and centrifuged for 10 min at 5000 x *g* at 4°C to separate the cells from matrix. The supernatant was filtered through a 0.2 μm cellulose acetate filter and the amount of eDNA was measured by using the QuantiFluor dsDNA System kit (Promega, Madison, WI, USA).

### 2.9 Statistical analysis

Unless otherwise specified, all statistical analysis was performed with a significance level of 0.05, using R 4.1.0.^20^ All MIC and MBEC values were transformed to log2 prior to analysis. Paired *t*‐tests were performed for metabolic activity, crystal violet and eDNA quantitation assay. For metabolic activity, crystal violet and eDNA quantitation assay for CF and non‐CF CRS isolates, one‐way ANOVA followed by Tukey's multiple comparison tests were performed by GraphPad Prism version 9.0.0 (Graph Pad Software, L.A Jolla, U.S).

Wilcoxon tests were performed to test whether there were differences in MIC and MBEC in aerobic and anaerobic conditions. For all 2‐way analyses investigating whether MBEC and MIC was affected by the interaction of aerobic and anaerobic growth conditions and other factor (including CF and non CF strains, and the country the strain was sourced from), a mixed effects ordinal logistic regression analysis was conducted using the ordinal R package.^21^

## 3 RESULTS

### 3.1 Planktonic *P. aeruginosa* grown in anaerobic condition exhibited increased gentamicin resistance

*P. aeruginosa* clinical isolates harvested from the sinonasal cavities of 25 patients were used in this study. These included 7 clinical isolates from CRS patients from The Netherlands (all CF), 11 isolates from CRS patients from Australia (2 CF and 9 non‐CF) and 7 CRS patients from USA (3 CF and 4 non‐CF).

**FIGURE 1:**
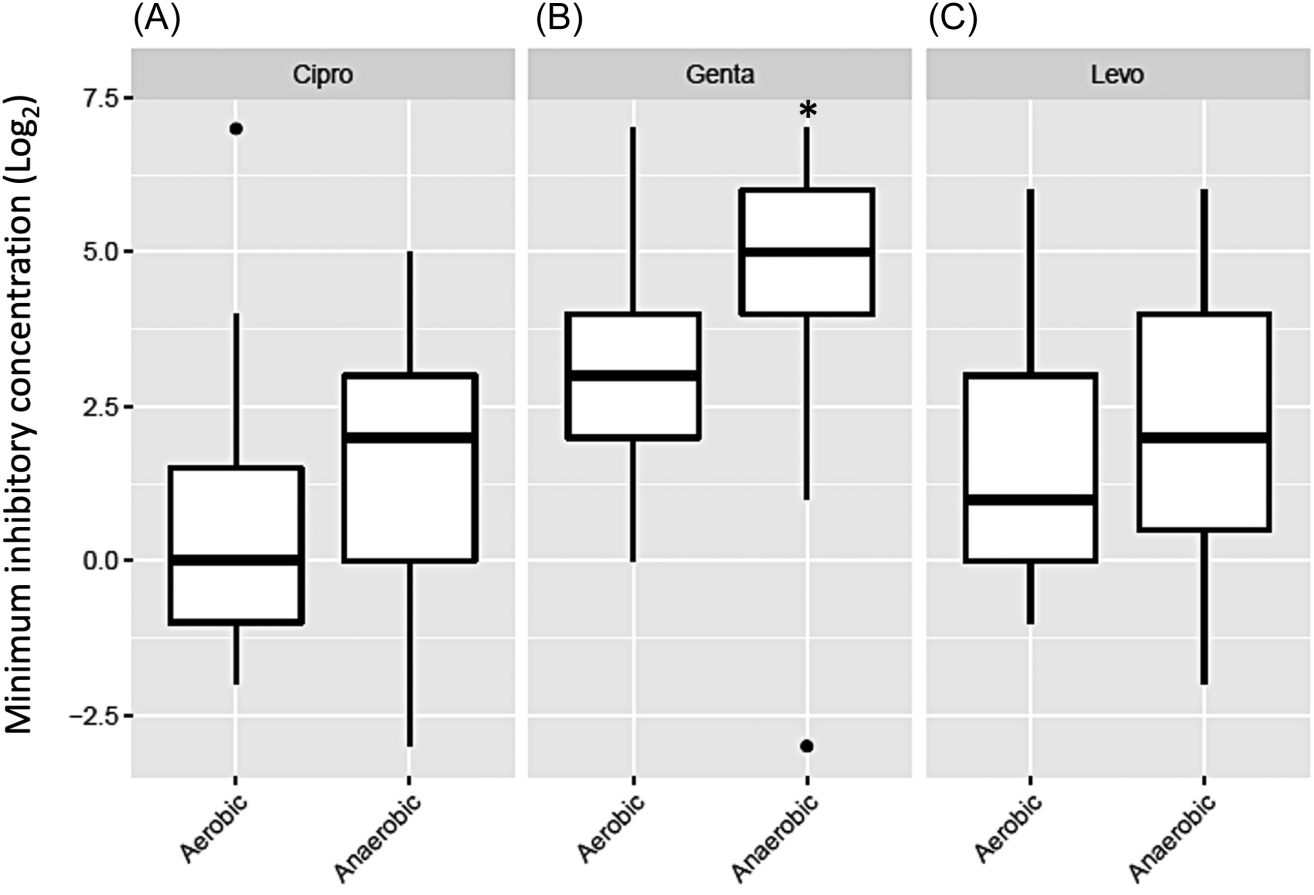
Comparison of minimum inhibitory concentrations (MIC) for all the *P. aeruginosa* isolates (*n* = 25) for three antibiotics: ciprofloxacin (A, Cipro), gentamicin (B, Genta) and levofloxacin (C, Levo). Average of log_2_ MIC data of isolates grown in aerobic and anaerobic conditions. Mixed effects ordinal linear regression, *p < 0.05.

Minimum inhibitory concentration (MIC) values of three antibiotics (ciprofloxacin, levofloxacin and gentamicin) were assessed for all isolates grown in aerobic and anaerobic conditions (Figure 1). In general, MIC values were not affected by the strain's country of origin or CF status (*p* > .05). Gentamicin showed a significant increase in MIC values when isolates were grown in anaerobic condition compared with aerobic condition (*p* = .04) (Figure 1B). MIC values for ciprofloxacin and levofloxacin for isolates grown in anaerobic conditions were higher compared to matched isolates grown in aerobic condition, however, statistical significance was not reached (*p* > .05) (Figure 1A,C).

### 3.2 *P. aeruginosa* isolates grown in biofilm form in anaerobic condition displayed increased MBEC values for gentamicin and levofloxacin than those grown in aerobic condition

**FIGURE 2:**
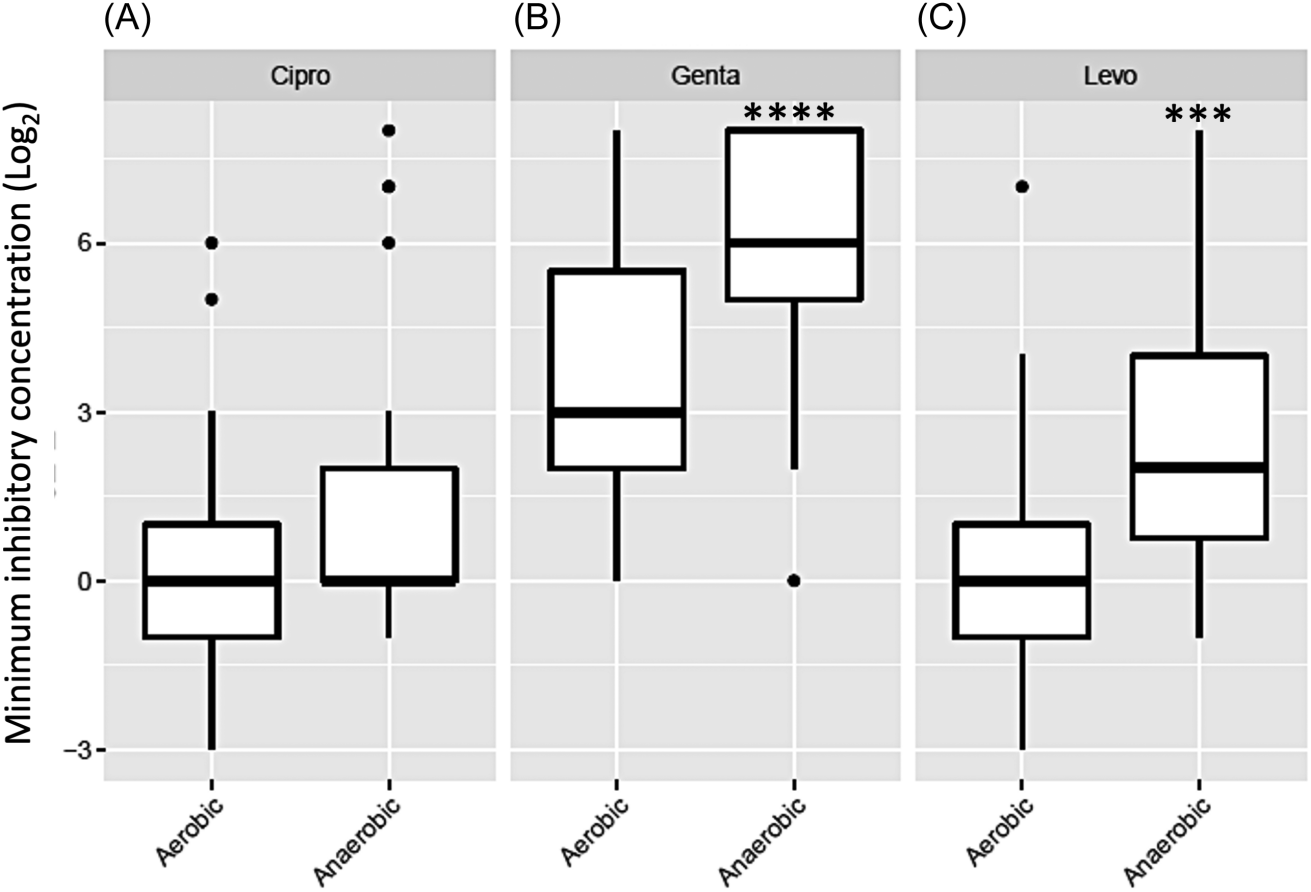
Comparison of minimum biofilm eradication concentrations (MBEC) for all the *P. aeruginosa* isolates (*n* = 25) for three antibiotics: ciprofloxacin (A, Cipro), gentamicin (B, Genta) and levofloxacin (C, Levo). Average of log_2_ MBEC data of isolates grown in aerobic and anaerobic condition. Mixed effects ordinal linear regression, *** *p* < 0.001; ****p < 0.0001.

**FIGURE 3:**
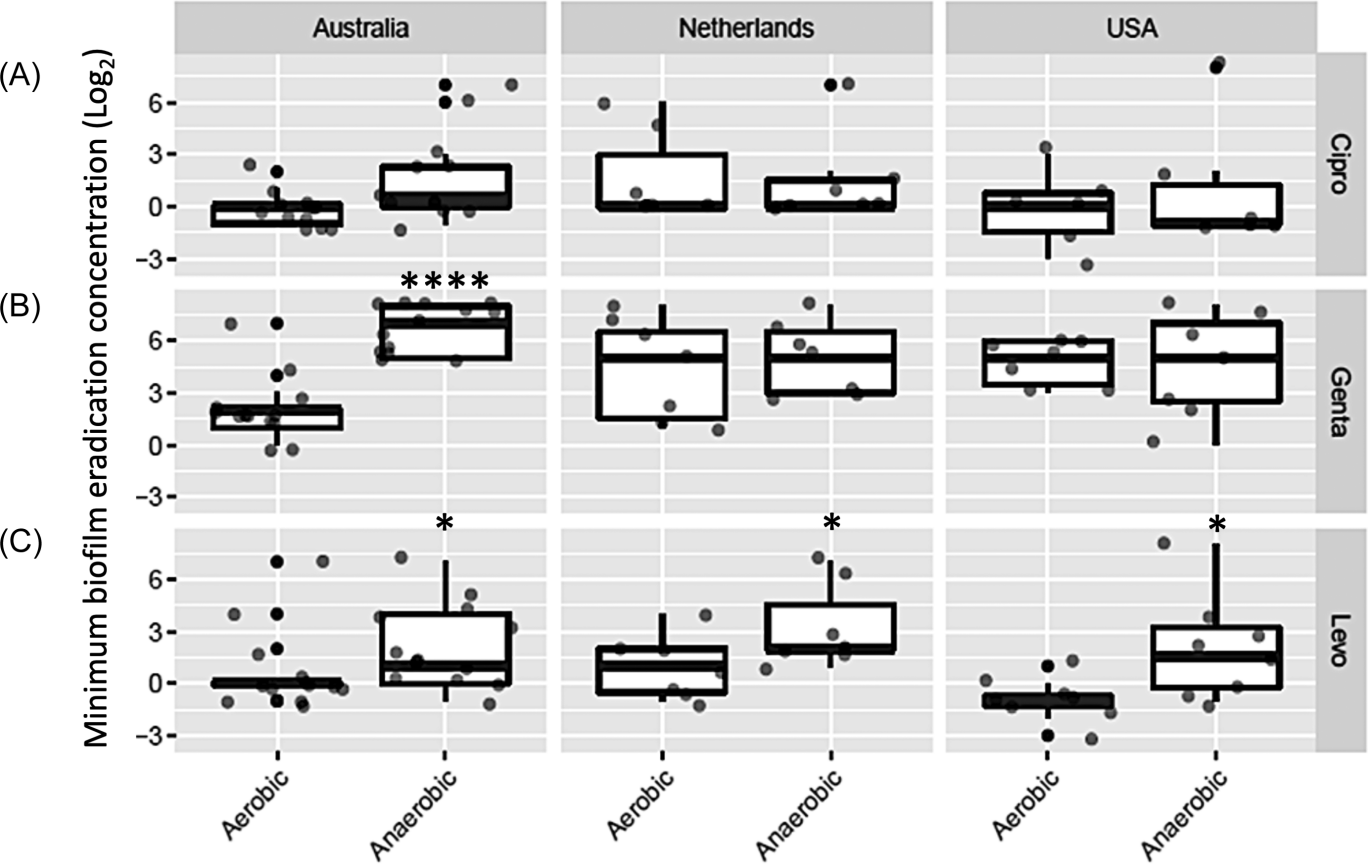
Comparison of minimum biofilm eradication concentrations (MBEC) for three antibiotics ciprofloxacin (A), gentamicin (B) and levofloxacin (C) in *P. aeruginosa* isolates from Australia, The Netherlands and USA (*n* = 25). Average of log_2_ MBEC data in aerobic and anaerobic conditions. Mixed effects ordinal linear regression, with dots indicating each sample. *p < 0–0.05;****p < 0.0001.

MBEC values were significantly higher for isolates grown in anaerobic condition compared to the same isolates grown in aerobic condition for gentamicin (*p* < .0001) and levofloxacin (*p* = .001) but not ciprofloxacin (*p* = .103) (Figure 2). Higher MBEC values for gentamicin were seen for Australian isolates grown in anaerobic condition compared to those isolates grown in aerobic condition (*p* = 3.07e‐06), whereas for the isolates from The Netherlands and USA, MBEC values for gentamicin were similar when grown in anaerobic and aerobic condition (Figure 3B). For levofloxacin, anaerobic MBECs were significantly higher than aerobic MBECs (*p* = .01) for isolates across the 3 countries (Figure 3C).

**FIGURE 4:**
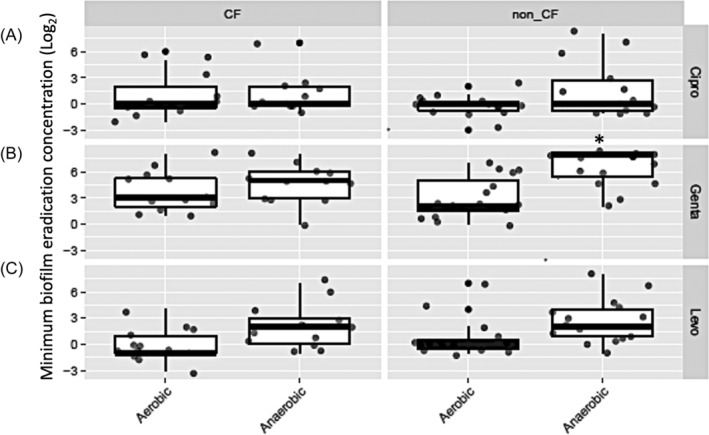
Comparison of minimum biofilm eradication concentrations (MBEC) for ciprofloxacin, gentamicin and levofloxacin in *P. aeruginosa* isolates from CF (*n* = 12) and non‐CF (*n* = 13) CRS patients grown in aerobic and anaerobic condition. Average of log_2_ MBEC data in aerobic and anaerobic conditions for CF and non‐CF patients. Mixed effects ordinal linear regression, with dots indicating each sample. *p < 0.05.

Further, we divided the CRS patients into non‐CF (*n* = 13) and CF (*n* = 12) groups. The difference in MBEC values between aerobic and anaerobic conditions was smaller in CF positive samples compared to non‐CF samples with a significant increase in MBEC values in gentamicin in non‐CF samples in anaerobic condition (*p* = .0159) (Figure 4).

### 3.3 The metabolic activity of anaerobic biofilms is significantly higher compared to aerobic biofilms

**FIGURE 5:**
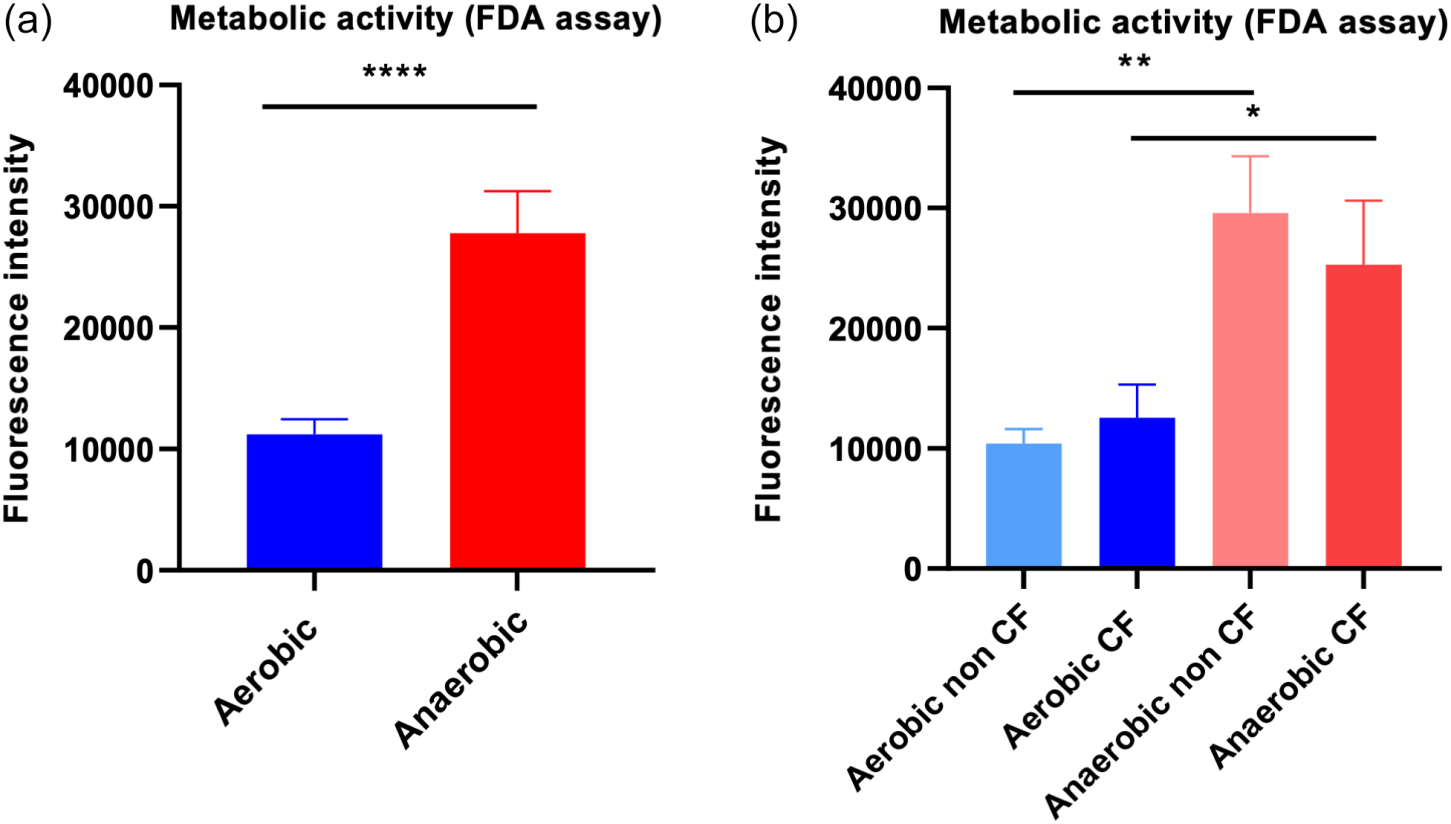
Metabolic activity of *P. aeruginosa* CRS isolates (*n* = 25) grown in biofilm form in aerobic and anaerobic conditions. Average fluorescence signals (measured by fluorescence intensity) obtained from biofilm of CRS patients (*n* = 25) grown in aerobic and anaerobic conditions (A). CRS patients divided into non‐CF (*n* = 13) and CF (*n* = 12) groups and the metabolic activity measured in aerobic and anaerobic conditions (B). One‐way ANOVA followed by Tukey's multiple comparison test, *p < 0.05, **p < 0.01, ****p < 0.0001.

FDA assays were carried out to determine the biofilm metabolic activity of isolates grown in aerobic and anaerobic condition. In general, the metabolic activity was significantly higher (*p* = .0001) for biofilms grown in anaerobic condition compared to those grown in aerobic condition (*n* = 20) (Figure 5A). Further, we divided the CRS patients into non‐CF (*n* = 11) and CF (*n* = 9) groups. As shown in Figure 5B, the CF condition did not significantly affect the metabolic activity of *P. aeruginosa* biofilms which was significantly increased in anaerobic condition compared to aerobic condition for both non‐CF and CF patients (*p* = .001 and *p* = .024, respectively) (Figure 5B).

### 3.4 The biofilm biomass of non‐CF isolates grown in aerobic conditions is higher compared to anaerobic conditions

**FIGURE 6:**
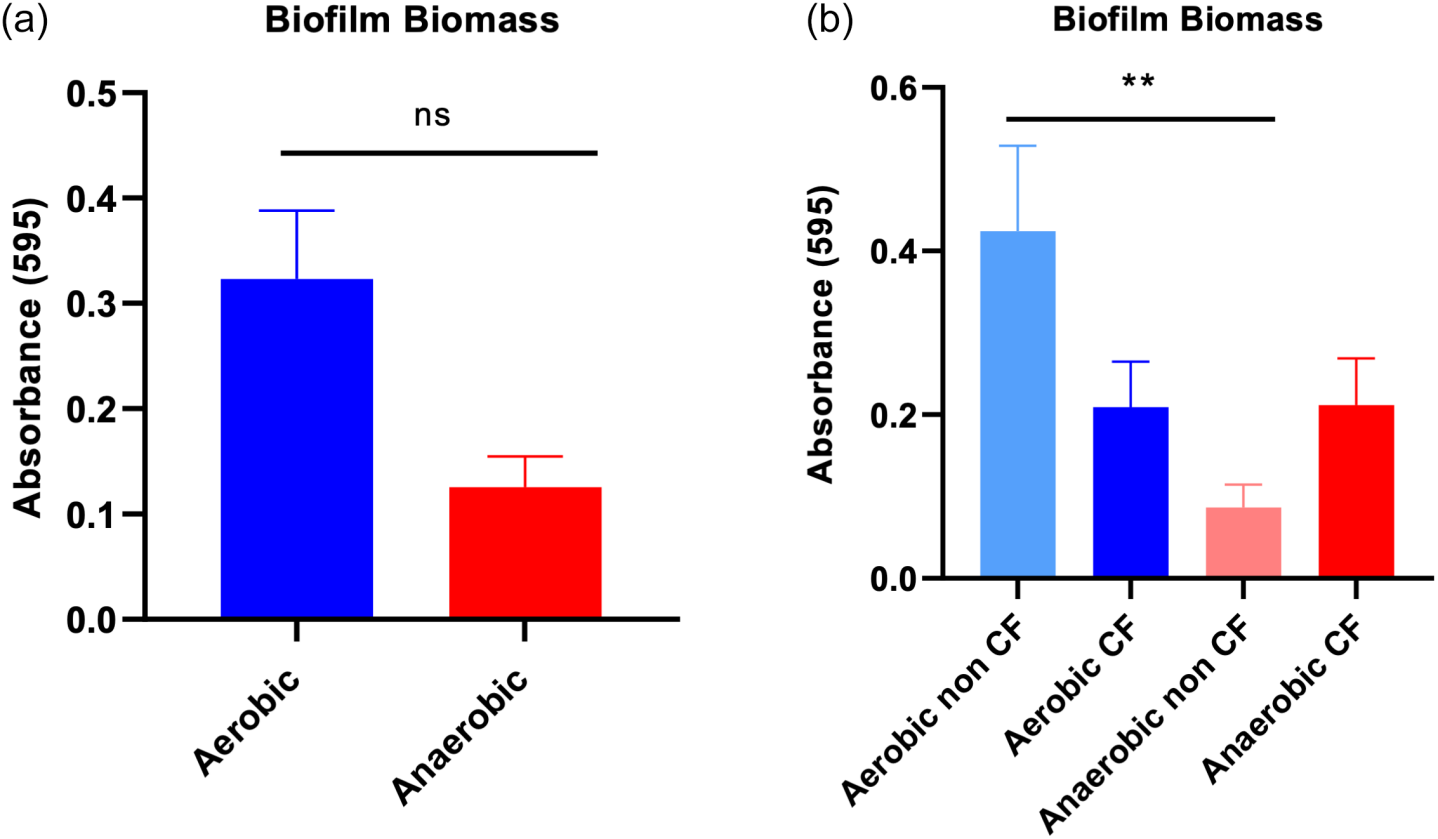
Crystal violet biofilm biomass of CRS isolates (*n* = 25) grown in aerobic and anaerobic conditions. Average absorbance obtained from biofilm of CRS patients (*n* = 25) grown in aerobic and anaerobic conditions (A). CRS patients divided into CF (*n* = 12) and non‐CF (*n* = 13) groups and the biomass yield measured in aerobic and anaerobic conditions (B). Simple paired *t*‐test (A) and one‐way ANOVA followed by Tukey's multiple comparison test (B), **p < 0.01.

The average absorbance values obtained from *P. aeruginosa* biofilms grown in aerobic and anaerobic conditions are shown in Figure 6A. In aerobic conditions, the biomass yield was higher compared with anaerobic conditions, although this did not reach statistical significance (*p* = .07). When the CRS patients (*n* = 25) were divided into non‐CF (*n* = 13) and CF (*n* = 12) groups, the biomass of aerobically grown biofilms of non‐CF isolates were significantly higher compared to those grown anaerobically (*p* = .001). In contrast, the biofilm biomass from CF patient isolates grown in aerobic and anaerobic conditions was similar (*p* = .999) (Figure 6B).

### 3.5 Extracellular DNA (eDNA) concentrations were significantly higher in aerobic conditions compared to anaerobic conditions

**FIGURE 7:**
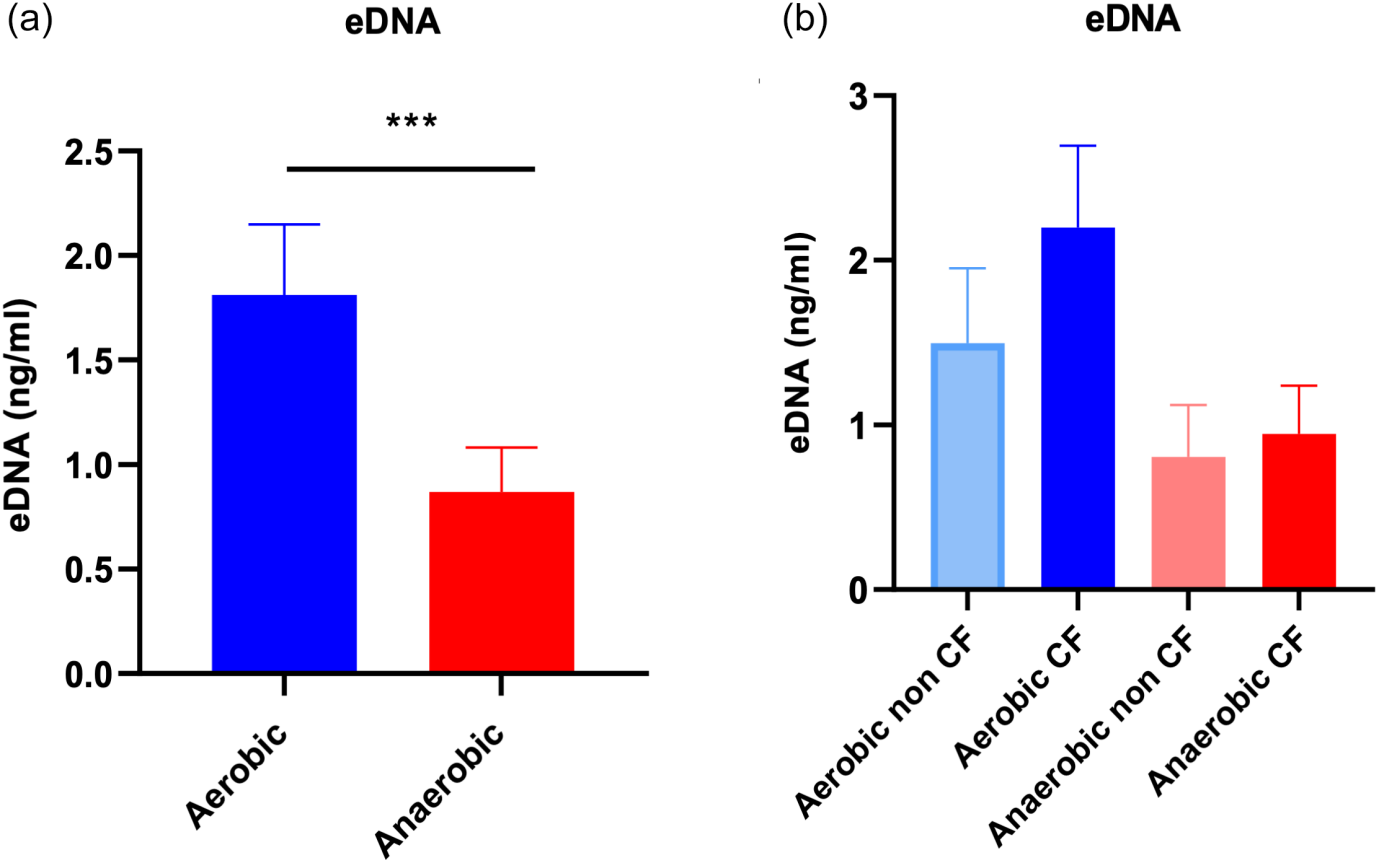
Extracellular DNA concentrations of CRS isolates in aerobic and anaerobic conditions (*n* = 20). Average of eDNA concentration in biofilm from CRS isolates (*n* = 25) in aerobic and anaerobic conditions (A). CRS patients divided into non‐CF (*n* = 13) and CF (*n* = 12) groups in aerobic and anaerobic conditions (B). Simple paired *t*‐test (A) and ordinary one‐way ANOVA followed by Brown‐Forsythe test (B). ***p < 0.001.

The amount of eDNA in *P. aeruginosa* biofilms of CRS isolates was significantly higher in aerobic than anaerobic condition (*p* < .001) (Figure 7A). After dividing the CRS patients (*n* = 25) into non‐CF (*n* = 13) and CF (*n* = 12) groups, eDNA levels were decreased in both conditions when grown under anaerobic conditions compared the same isolates grown under aerobic conditions. However, this did not reach statistical significance (Figure 7B).

## 4 DISCUSSION

In this study, we compared the MIC and MBEC values of three commonly used antibiotics (ciprofloxacin, levofloxacin and gentamicin) in 27 *P. aeruginosa* isolates from CRS patients grown in aerobic and anaerobic conditions. The MIC values for gentamicin and the MBEC values for levofloxacin and gentamicin were significantly higher for isolates grown in anaerobic conditions compared to the same isolates grown in aerobic conditions. In a country‐wise analysis, some variability was seen with MBEC values between countries. Namely, MBEC values were significantly higher for gentamicin in Australian isolates grown in anaerobic conditions compared to the same isolates grown in aerobic conditions, however, those differences were not seen for isolates from the US and The Netherlands. In contrast, levofloxacin showed significantly higher MBEC values in anaerobic conditions among all three countries. Our study also demonstrated that the metabolic activity in anaerobically cultured biofilms was significantly higher compared to aerobic biofilms. In contrast, eDNA concentrations and biofilm biomass were higher in aerobic conditions compared to anaerobic conditions even though the latter was seen for the non‐CF isolates only.

Anaerobic growth and biofilm formation both play significant roles in antibiotic resistance and it is well known that bacteria growing in biofilms are much more tolerant to antibiotics than their planktonic counterparts.^9^ In line with our results, several studies have shown that growth in anaerobic conditions of *P. aeruginosa* increases their antibiotic resistance even though some variability is seen for various antibiotics.^14^, ^22^ For example, oxygen limitation has been shown to contribute to 62% resistance for ciprofloxacin, 69% for tobramycin, 80% for tetracycline and >110% for ceftazidime.^14^ It has been proposed that anaerobic growth conditions induce changes in the structure of the outer membrane of *P. aeruginosa*, resulting in the loss of the O‐specific antigens (formerly B‐band polysaccharides).^23^, ^24^ Furthermore, the outer membrane affinity for gentamicin was higher in *P. aeruginosa* strains possessing the B band than in strains with A band LPS.^25^ Strains which are missing O‐specific antigens have indeed been shown to be more tolerant to gentamicin^25^ and this could at least in part explain the increased resistance of the tested strains to gentamicin when grown in anaerobic condition identified in this study. In our study, not all tested antibiotics had increased MIC and MBEC values in anaerobic state compared to those grown in aerobic condition. This was also seen by others where Davey et al.^13^ reported that ciprofloxacin MIC and MBEC were reduced for *P. aeruginosa* grown in anaerobic conditions but the MIC and MBEC for gentamicin increased significantly when isolates were grown in anaerobic conditions. Notably, the increased gentamicin MBEC values in anaerobic condition were seen in this study only for Australian isolates and not for isolates from the USA and The Netherlands. Differences in antibiotics usage in the various countries could contribute to this. Additionally, the differences in MBEC values for the various antibiotics for isolates grown in aerobic and anaerobic condition were smaller for those isolates sourced from CF CRS patients compared to those harvested from non‐CF CRS patients. This might reflect the adaptation of CF CRS patient sourced isolates to their environment where frequent antibiotics exposure and anaerobic conditions in the upper airways of CF patients are common.^12^ Furthermore, this might also contribute to the increased MBEC values for gentamicin for Australian isolates as most of those isolates were from non‐CF patients.

This study found that the increased MBEC values for anaerobically grown biofilms were accompanied by an increased metabolic activity and a decrease in biomass and eDNA content. This is contrary to what would reasonably be expected as both a reduction in metabolic activity and the presence and thickness of an extracellular polymeric substances (EPS) matrix and thus biomass of biofilms are thought to be important contributors to the antibiotic resistance of biofilms.^26^ Under low oxygen conditions in biofilms and in CF airways, *P. aeruginosa* can grow anaerobically by utilizing the alternative electron acceptors NO_3_− and NO_2_− and thus in our study, anaerobic growth was achieved by the addition of nitrates to the media.^8^ It has been shown that the combination of local oxygen deficiencies and the presence of nitrate contribute to the reduced susceptibility to various antibiotics.^14^ Furthermore, in a model of alginate‐encapsulated *P. aeruginosa*, creating diffusion limitations through the secondary matrix, as seen in chronic infections, supplementation with NO3^−^, could alleviate electron acceptor limitation, thereby stimulating metabolism.^27^ Hence, nitrate supplementation in oxygen limited conditions is likely to play an important role in metabolic activation and antibiotic resistance. A limitation of this study is the lack of healthy control patients. Future considerations may involve exploring the isolation of *Pseudomonas* from non‐CRS patients to assess whether their resistance profile differs, potentially influenced by the mucosa's exposure to presumably more normoxic conditions.

In conclusion, our results provide an understanding of the antibiotic resistance of *P. aeruginosa* in planktonic and biofilm form in isolates from CRS patients with and without CF in aerobic and anaerobic conditions. These findings might help to support a rationale for further in vivo studies aimed at investigating the optimal antibiotic and dosage to be used for the treatment of chronic *P. aeruginosa* infections where anaerobic conditions exist.

## CONFLICT OF INTEREST STATEMENT

The authors declare no conflicts of interest that are relevant to this paper.
